# Airway pressure release ventilation

**DOI:** 10.4103/1817-1737.36556

**Published:** 2007

**Authors:** Ehab G. Daoud

**Affiliations:** *Department of Critical Care Medicine, The Miriam Hospital, Brown University, Providence, Rhode Island, USA*

**Keywords:** Critical care, mechanical ventilation, respiratory failure

## Abstract

Airway pressure release ventilation was introduced to clinical practice about two decades ago as an alternative mode for mechanical ventilation; however, it had not gained popularity until recently as an effective safe alternative for difficult-to-oxygenate patients with acute lung injury/ acute respiratory distress syndrome This review will cover the definition and mechanism of airway pressure release ventilation, its advantages, indications, and guidance.

## Introduction

Airway pressure release ventilation (APRV) was introduced to clinical practice about two decades ago as an alternative mode for mechanical ventilation; however, it had not gained popularity until recently as an effectivesafe alternative for difficult-to-oxygenate patients with acute lung injury/ acute respiratory distress syndrome (ALI/ARDS). APRV has many appealing features applicable to our current understanding of ALI/ ARDS treatment, such as minimizing ventilator-induced lung injury (VILI) using lung protective strategies. There have been only few studies on APRV, mostly on animals and even fewer on humans, some showing superiority to the conventional ventilatory methods but none showing any mortality differences. In this review, we will answer four important questions about APRV:What is it (definition and mechanism of action)? Why use it (advantages) When to use it (indications and contraindications)How to use it (guidelines and troubleshooting)

## What is airway pressure release ventilation (APRV)

**Figure 1:**
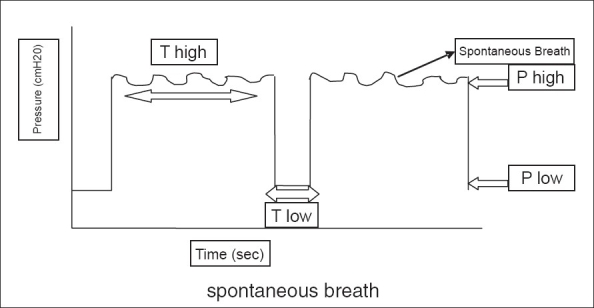
Pressure-time curve for APRV. 'P high' is the high CPAP, 'P low' is the low CPAP, 'T high' is the duration of 'P high,' and 'T low' is the release period or the duration of 'P low.' Spontaneous breathing appears on the top of 'P high.'

APRV was described initially by Stock and Downs in 1987[1,2] as a continuous positive airway pressure (CPAP) with an intermittent release phase. APRV applies CPAP (P high) for a prolonged time (T high) to maintain adequate lung volume and alveolar recruitment, with a time-cycled release phase to a lower set of pressure (P low) for a short period of time (T low) or (release time) where most of ventilation and CO_2_ removal occurs [Figure 1].

Using high-flow (demand valve) CPAP circuit, unrestricted spontaneous breathing can be integrated and can happen any time regardless of the ventilator cycle. If the patient has no spontaneous respiratory effort, APRV becomes typical to ‘inverse ratio pressure’-limited, ‘time cycle’-assisted mechanical ventilation (pressure-controlled ventilation).[3]

In acute respiratory distress syndrome (ARDS), the functional residual capacity (FRC) and lung compliance are reduced, and thus the elastic work of breathing (WOB) is elevated. By applying CPAP, the FRC is restored and inspiration starts from a more favorable pressure-volume relationship, facilitating spontaneous ventilation, and improves oxygenation.[4] Applying ‘P high’ for a ‘T high’ (80–95% of the cycle time), the mean airway pressure is increased insuring almost constant lung recruitment (open-lung approach), in contrast to the repetitive inflation and deflation of the lung using conventional ventilatory methods, which could result in ventilator-induced lung injury (VILI);[5,6] or the recruitment maneuvers, which have to be done frequently to avoid derecruitment. Mean airway pressure on APRV is calculated by this formula: $\frac{\text{(P High }\times \text{ T High) + (P Low }\times \text{ T Low)}}{\text{(T High + T Low)}}$

Minute ventilation and CO_2_ removal in APRV depend on lung compliance, airway resistance, the magnitude and duration of pressure release and the magnitude of the patient's spontaneous breathing efforts. Spontaneous breathing plays a very important role in APRV, allowing the patient to control his/ her respiratory frequency without being confined to an arbitrary preset inspiratory:expiratory ratio (I:E), thus improving patient comfort and patient-ventilator synchrony with reduction of the amount of sedation necessary. Additionally, spontaneous breathing helps drive the inspired gas to the nondependent lung regions by using the patient's own respiratory muscles and through pleural pressure changes without raising the applied airway pressure to a rather dangerous level, as in conventional mechanical ventilation, producing more physiological gas distribution to the nondependent lung regions and improving ventilation/ perfusion (V/ Q) matching.[7–10]

The addition of pressure support ventilation (PSV) above ‘P high’ to aid spontaneous breaths is feasible, but this addition contradicts limiting the airway pressure and may cause significant lung distention; furthermore, the imposition of PSV to APRV reduces the benefits of spontaneous breathing by altering the normal sinusoidal flow of spontaneous breath to a decelerating assisted mechanical breath as flow and pressure development are uncoupled from patient effort. Ultimately, PSV during APRV defeats its purpose and is not recommended.[11–13] On the other hand, the use of automatic tube compensation (ATC) during APRV may help overcome the artificial airway resistance during spontaneous breathing using computerized ventilator algorithms without causing overt lung distention while preserving the sinusoidal flow pattern of spontaneous breath.[14]

## Why use APRV (advantages)

### I. Effects on oxygenation

The improved oxygenation parameters (PaO_2_ / FiO_2_, lung compliance) during APRV are attributed to the beneficial effects of spontaneous breathing through better gas distribution and better V/Q matching to the poorly aerated dorsal region of the lungs, along with higher mean airway pressure obtained compared to conventional ventilation: ‘open lung approach.’

Multiple studies in humans and animals have shown improved oxygenation, better V/Q match and lesser dead space compared to conventional mechanical ventilation;[2,7,9–11] other studies have shown no significant differences in oxygenation parameters, but with significantly less applied pressures and adverse effects than conventional mechanical ventilation.[15–18]

### II. Effects on hemodynamics

During spontaneous breathing, the pleural pressure decreases, leading to a decrease in the intra-thoracic and right atrial pressure – thus increasing venous return and improving the pre-load and consequently increasing the cardiac output.[3] Kaplan *et al.*[19] compared the hemodynamic effects in patients with ALI/ ARDS on APRV versus inverse ratio PCV; they found significantly higher cardiac index (CI l/min/m^2^)), oxygen delivery (DO_2_ ml/min), mixed venous oxygen saturation (SVO_2_ %), urine output (ml/kg/h) and significantly lower vasopressors and inotropes usage, lactate concentration (mmol/L) and CVP (mmHg) while on APRV. Putnsen *et al.*[7] compared APRV and PCV in 30 trauma patients, and they found significantly less vasopressors and positive inotropes usage, with significantly increased CI, DO_2_.

### III. Effects on regional blood flow and organ perfusion

In a study by Hering *et al.,*[20] APRV improved respiratory muscle blood flow in 12 pigs with ALI. In a similar study by the same authors,[21] APRV showed improved blood flow to stomach, duodenum, ileum and colon in 12 pigs with ALI. Kaplan *et al.*[19] found significantly improved urine output and glomerular filtration rate in patients on APRV as compared to PCV.

### IV. Effects on sedation and neuromuscular blockades usage

The level of analgesia and sedation required during CMV is usually equivalent to a Ramsay score of between 4 and 5 (i.e., a deeply sedated patient) during APRV; a Ramsay score of between 2 and 3 can be targeted (i.e., an awake patient who is responsive and cooperative).

APRV had shown to decrease the need for neuromuscular blockades use by 70% and the use of sedation by about 40% compared to conventional mechanical ventilation in other studies.[7,10,16,18,19] The decreased usage of sedatives and neuromuscular blockers may translate into decreased length of mechanical ventilation and ICU length of stay.[7,22]

## When to use APRV

### Indications

Based on clinical and experimental data, APRV is indicated in patients with ALI, ARDS and atelectasis after major surgery.[8,9,11,17,19,20]

### Contraindications

Because of the lower levels of sedation used to allow spontaneous breathing, APRV should not be used in patients who require deep sedation for management of their underlying disease (e.g., cerebral edema with increased intracranial pressure or status epilepticus).

To date, no data are available on the use of APRV in patients with obstructive lung disease (bronchial asthma exacerbations or chronic obstructive pulmonary diseases). Theoretically, using short release time is not beneficial in those patients who require prolonged expiratory time.

Likewise, use of APRV has not been investigated in patients with neuromuscular disease and is not supported by any evidence.

## How to adjust pressures and tidal volumes during APRV

Mechanical ventilation with positive end-expiratory pressure (PEEP) titrated above the lower inflection point of the static pressure-volume curve and a low tidal volume at 6 ml/kg (TV) are thought to prevent alveolar collapse at end-expiration and over distension of lung units at end-inspiration in patients with ARDS. This lung-protective strategy has shown improvement in mortality in patients with ARDS.[23,24]

The setup at the bedside is simple and the goals are the same: to maintain adequate oxygenation and ventilation without overt lung distention during ‘P high’ and avoiding lung derecruitment and/ or intrinsic PEEP during ‘P low.’

### Setting pressures:

**Figure 2:**
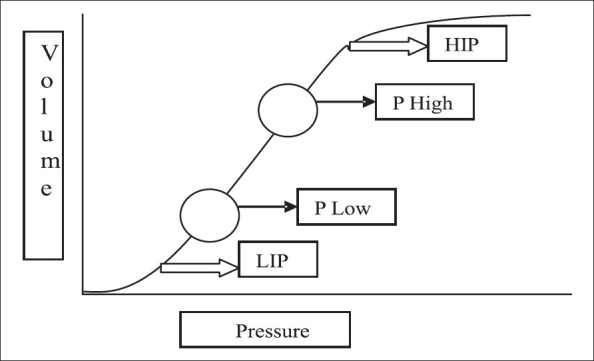
Static pressure-volume curve during volume-controlled mechanical ventilation. High pressure ('P high') is set below the high inflection point (HIP) and low pressure is set above the low inflection point (LIP).

‘P high’ should be below the high inflection point (HIP) on the static volume-pressure curve, while ‘P low’ should be above the low inflection point (LIP) on the same curve [Figure 2].

### Setting times:

**Figure 3:**
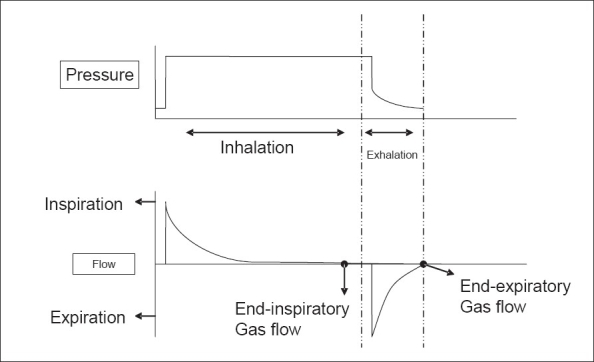
Corresponding pressure and flow curves during one cycle of inflationdeflation. Notice the flow curve goes back to zero at the end of inflation, indicating full lung inflation; and also goes back to zero during the release period before inflation starts, indicating complete gas exhalation with no intrinsic PEEP.

‘T high’ should allow complete inflation of the lungs, as indicated by an end-respiratory phase of no flow when spontaneous breathing is absent, and ‘T low’ should allow for complete exhalation with no gas flow at its end to assure absence of intrinsic or auto PEEP [Figure 3].

It is also recommended setting ATC to 100% and trying to avoid over-sedation.

Initial setup and transition from conventional ventilation

P high is usually set at a level between 20 and 30 cm H_2_O.

P low is set between 0 and 5 cm H_2_O initially.

T high: 4–6 s

T low: 0.2-0.8 seconds

## Troubleshooting

- Maneuvers to correct poor oxygenation include 1) increase either ‘P high,’ ‘T high’ or both to increase mean airway pressure; 2) change the patient position to the prone position along with APRV.[3]
- Maneuvers to correct poor ventilation include 1) increase ‘P high’ and decrease ‘T high’ simultaneously to increase minute ventilation while keeping stable mean airway pressure (preferred method); 2) increase ‘T low’ by 0.05-0.1 s increments; 3) decrease sedation to increase the patient's contribution to minute ventilation.[3]

## Conclusions

APRV is a simple, safe and effective ventilatory method for patients with ALI/ ARDS; currently there is some but no strong evidence to suggest its superiority above other ventilatory methods in regard to oxygenation, hemodynamics, regional blood flow, patient comfort and length of mechanical ventilation. There is no evidence of improved mortality outcome by using APRV as compared to other modes of mechanical ventilation. There is a need for large human trials to compare APRV to conventional mechanical ventilation using lung-protective strategies before drawing final conclusions about this interesting mode of ventilation. Currently we do not recommend APRV for every patient with ALI/ ARDS; but for carefully selected patients, consultation with specialist and respiratory therapist with expertise in using APRV may be necessary.
